# Genomic profiling and immune landscape of olfactory neuroblastoma in China

**DOI:** 10.3389/fonc.2023.1226494

**Published:** 2023-11-01

**Authors:** Yunyun Yang, Zhiyi Wan, Enli Zhang, Yingshi Piao

**Affiliations:** ^1^ Department of Pathology, Beijing Tongren Hospital Affiliated to Capital Medical University, Beijing, China; ^2^ Department of Medicine, Beijing Key Laboratory of Head and Neck Molecular Diagnostic Pathology, Beijing, China; ^3^ Department of Medicine, Genecast Biotechnology Co., Ltd., Wuxi, China

**Keywords:** genomic, immune landscape, olfactory neuroblastoma, tumor, prognosis

## Abstract

**Background:**

Olfactory neuroblastoma (ONB) is a rare malignant neoplasm of the olfactory mucosa. The paucity of genomic data has prevented the development of individualized ONB treatments. Here, we investigated the genomic and immune landscape of ONB in Chinese patients.

**Methods:**

Whole exome sequencing (WES) and multiplex immunofluorescence (MIF) analysis were performed on tissue samples from 19 Chinese ONB patients. Patients were divided into low- and high-grade groups.

**Results:**

Overall, 929 nonsynonymous alterations were identified in 18 (94.74%) ONB cases. The most prevalent altered cancer-related genes were *CTNNB1* (16%) and *ZNRF3* (16%). The most mutated oncogenic pathways were the WNT and RAS pathways. The median tumor mutation burden (TMB) was 0.45, ranging from 0 to 3.25. Only one case expressed PD-L1 (> 1%) in the tumor region. The percentage of CD8+ tumor-infiltrating lymphocytes (TILs) in the tumor region ranged from 0.03% to 84.9%, with a median of 1.08%. No significant differences were observed between the low- and high-grade groups for clinicopathological features, mutant genes, mutant pathways, TMB, tumor neoantigen burden (TNB), mutant-allele tumor heterogeneity (MATH), PD-L1 expression levels, or CD8+ TIL percentage. However, the low-grade group showed significantly more CD68+ macrophages in both the tumor and total region than the high-grade group. Notably, CD68+CD163- macrophages accounted for an average of 80.5% of CD68+ macrophages.

**Conclusion:**

This study presents data on the genomic and immune landscape of ONB cases in China. *CTNNB1* and *ZNRF3* were the most prevalent altered cancer-related genes. The results of TMB, PD-L1, and CD8+ Tils suggest that ONB may be insensitive to immunotherapy. M1 macrophages may be positively associated with the prognosis of ONB.

**Implications for Practice:**

In this study, the most prevalent altered cancer-related genes were *CTNNB1* (16%) and *ZNRF3* (16%). The most mutated oncogenic pathways were the WNT and RAS pathways. The median tumor mutation burden (TMB) was 0.45, ranging from 0 to 3.25. Only one (1/15) case expressed PD-L1 (> 1%) in the tumor region. However, the low-grade group showed significantly more CD68+ macrophages in both the tumor and total region than the high-grade group. The higher level of CD68-related macrophages indicates that M1 macrophages potentially play an important role in ONB development that is possibly associated with prognosis.

## Introduction

Olfactory neuroblastoma (ONB) is a rare sinonasal neoplasm believed to arise from the olfactory neuroepithelium (1). Historically, the nodal and metastatic status and parameters related to tumor extensions, such as the Kadish staging system and Dulguerov classification, have been considered as the main prognostic factors (2). However, both the Kadish and Dulguerov classification systems are poorly informative of tumor aggressiveness, and their prognostic value has been challenged by several reports (3). Recently, Hyams’ grade has emerged as a critical factor for treatment planning. High-grade tumors (especially grade four) have a higher propensity for metastasis and may require more intensive treatments, whereas low-grade lesions can be managed with less aggressive therapeutic protocols (4, 5). However, it is difficult to diagnose ONB, especially high grade ONB. The majority of sinonasal and skull base regions tumors are poorly or undifferentiated tumors manifesting overlapping features that result in diagnostic challenges. Sinonasal neuroendocrine carcinoma, sinonasal undifferentiated carcinoma and ONB, share overlapping clinical, radiological, and histopathological features, albeit with variability in behavior and prognosis between each other (6). In this study, we investigated the genomic features of ONB in China and compared them between low- and high-grade ONB cases.

The rarity of ONB has limited the scope of clinical research studies, particularly those investigating the genetic and biochemical processes driving tumorigenesis. Only a few studies have applied DNA sequencing techniques to profile ONB, and their findings were not consistent. They reported few recurrent genomic aberrations or somatic mutations in known cancer genes (6, 7). One study found complex karyotypic disturbances that led to the amplification of *FGFR1*, *FANCC*, *NOTCH1*, *CBFA2T3*, *RXRA*, *NSMAF*, and *ASPH* (8). Another study recognized eight candidate cancer genes in ONB: *BRINP1*, *CARD11*, *CDKN2C*, *MEIS1*, *MINK1*, *PPP6C*, *TGFBR2*, and *TP53* (9). No mutations in ONB tumors are reported in commonly used databases that collate genomic cancer data, such as COSMIC, cBioPortal, and ICGC portal (7). To date, no genomic studies of ONB have been conducted in China.

This paucity of genomic data, in turn, has in some ways limited the discovery of new therapeutic strategies for ONB. The current treatment approach centers on surgical resection in combination with radiotherapy and/or chemotherapy, as needed (10). However, these multimodality nontargeted therapies for relapsed ONB are of limited clinical benefit. Studies on the immune landscape include analyses on PD-L1 expression and analysis on TILs led to breakthrough trials of programmed death-1 (PD-1) inhibitors for recurrent/metastatic head and neck squamous cell carcinoma therapy (11). However, the role of PD-L1 in ONB is unclear and has been investigated in few studies with contradictory results (12). PD-L1 expression and lymphocyte distribution in Chinese ONB patients need to be elucidated.

In the present study, we performed whole exome sequencing (WES) on 19 clinical ONB samples from China. Examining the genomic landscape of ONB may help clarify the biological mechanisms underlying this tumor type and identify potential treatment targets. Moreover, we characterized the immune landscape of ONB, which may provide critical details for future research.

## Methods

### Patients and clinical specimens

We retrospectively investigated 19 patients with ONB profiled at Beijing Tongren Hospital between May 2018 and October 2020. Sections from formalin-fixed paraffin-embedded (FFPE) tissue sections were stained with hematoxylin and eosin (H&E) and examined by experienced pathologists to confirm the pathological diagnosis and ensure that the tumor content was ≥ 20%. The grade relies on a four-tier grading scale based on lobular architecture, mitosis, nuclear pleomorphism, neurofibrillary matrix, necrosis, calcifications, rosettes and pseudo-rosettes. It is complex to use, especially in borderline cases encompassing grade III and IV.in order to correlate the grade with outcomes, many studies tend to separate ONB into low-grade (Hyams I and II) and high-grade (Hyams III and IV) lesions thereby hampering optimal clinical management (13). This study was conducted with the approval of the Ethics Committee of Beijing Tongren Hospital (No. MR-11-23-000050), and informed consent was obtained from all patients. Data for French ONB patients were from a previous report (14). The diagnosis of low and high grade ONB was based only on H&E staining. We were sure that all 19 cases are true ONB, and 17/19 cases primary ONB and 2/19 recurrent ONB, 7 ONB patients were low grade and 12 ONB patients were high grade.

### DNA extraction

DNA was extracted from FFPE specimens using the QIAamp DNA FFPE Tissue Kit (Qiagen, Hilden, Germany) according to the manufacturer’s protocol. The quantity and quality of the isolated DNA were assessed using a Qubit 3.0 fluorimeter (Thermo Fisher Scientific, Waltham, MA, USA).

### WES analysis

Library preparations were performed using the Twist Human Core Exome Kit (Twist Bioscience, South San Francisco, CA, USA) according to the manufacturer’s protocol. The 150 bp paired-end sequencing was performed on an Illumina NovaSeq 6000 platform (Illumina, San Diego, CA, USA) according to the manufacturer’s protocol.

Sequenced sequences were first filtered using fastp (15) and then aligned to the reference genome (hg19) using Burrows-Wheeler Aligner (BWA) (16). Single nucleotide variants (SNVs) and small insertions and deletions (InDels) were identified by Mutect2 and TNseq (17), and were annotated using ANNOVAR (18). Finally, somatic mutations were excluded using the following criteria: (1) located in intergenic regions or intronic regions; (2) synonymous SNVs; (3) allele frequency < 3%; (4) support reads ≤ 3; (5) frequency ≥ 1% in the Genome Aggregation Database (gnomAD) and Exome Aggregation Consortium (ExAC) (19, 20); (6) assessed as ‘tolerated’ by SIFT or ‘benign’ by PolyPhen. Copy-number variations (CNVs) were called using CNVkit (21). The mutant-allele tumor heterogeneity (MATH) score was calculated with the formula: 100 × median absolute deviation/median of the Variant allele fraction (VAF) (22). The tumor mutation burden (TMB) was determined as the number of nonsynonymous somatic mutations per megabase (Mb) (23). Human leukocyte antigen typing was performed using HLA-HD (24). The tumor neoantigen burden (TNB) was calculated using pVACtools (25). A cancer-related gene was defined as “Oncogene” and “Tumor suppressor gene” in the OncoKB database.

### Multiplex immunofluorescence analysis

FFPE sections (4 μm-thick) were obtained from each sample. MIF staining was performed at Genecast Biotechnology Co., Ltd. (Beijing, China) as previously described (26). Briefly, slides were deparaffinized, rehydrated, and subjected to antigen retrieval. Endogenous peroxidase and protein blocking were then performed using Antibody Diluent/Block (#72424205, PerkinElmer, Waltham, MA, USA) for 10 minutes. One antigen was stained in each round, including primary antibody incubation, secondary antibody incubation, and tyramine signal amplification (TSA) visualization. The next antigen was then stained after epitope retrieval and protein blocking as before. The primary antibodies used were as follows: CD8 antibody (1:100, ZA0508, Zsbio, China), CD68 antibody (1:100, ZM0060, Zsbio), CD163 antibody (1:100, ab189915, Abcam, Cambridge, UK), PD-1 antibody (1:50, ZM0381, Zsbio), and PD-L1 antibody (1:25, ZA-0629, Zsbio). Nuclear counterstaining was performed using 4’,6-Diamidino-2-Phenylindole (DAPI). Slide images were acquired and analyzed using the PerkinElmer Vectra (V.3.0.5) and inForm V.2.3.0 software (PerkinElmer). We calculated the percentage of positive cells in 15 of fields of view per slide. The infiltration levels of positively stained cells were further determined by the percentage of positive cells in the tumor, stroma, and total region, respectively.

All cases were examined using the immunohistochemical En vision method with DAB staining. Antibodies for Ki-67 were purchased from Beijing Zhong Shan Jinqiao Biotechnology Company. All procedures were conducted according to the reagent instructions with appropriate negative and positive controls (27). The Ki-67 labeling index (Ki-67 LI) was determined at a magnification of 400x. The highest Ki-67 LI was determined by calculating the percentage of positive tumor cell nuclei within the three fields with the highest apparent number of stained nuclei.

### Statistical Analysis

Statistical analysis was performed using SPSS 22.0 statistical software. Fisher’s Exact test was used to compare the rates between groups with different characteristics. Wilcoxon test was employed to examine the differences in TMB, TNB, MATH, and immune cell proportions between the low- and high-grade groups. Statistical tests were two-sided and *P* < 0.05 was considered significant.

## Results

### Mutational spectrum of Chinese ONB patients

A total of 19 matched ONB tumor and normal samples were successfully examined by WES, yielding a mean depth of 305× and mean uniformity of 99.54%. The median patient age at diagnosis was 42 years (range: 22–72 years), with samples from 11 men and 8 women. Detailed clinicopathological information is listed in **Table S1**. The low- and high-grade groups did not show significant differences in most clinicopathological features, including age, gender, and Kadish stage (**Table 1**). Ki-67 expression in the high-grade group was significantly higher than that in the low-grade group which was consisted with previous study suggest that the Ki67 could be used as prognostic markers, as a potential alternative to the Hyams’ grade.

**Table 1:** Clinical characteristics of patients.

Clinical features	Hyams’ grade		P value
	Low group	High group	
Gender			1
Male	4	7	
Female	3	5	
Age (years)			0.377
≥ 45	4	4	
< 45	3	8	
Kadish			0.333
AB	1	5	
C	6	7	
Ki-67			0.045
>25	2	10	
<25	5	2	

**Figure 1:**
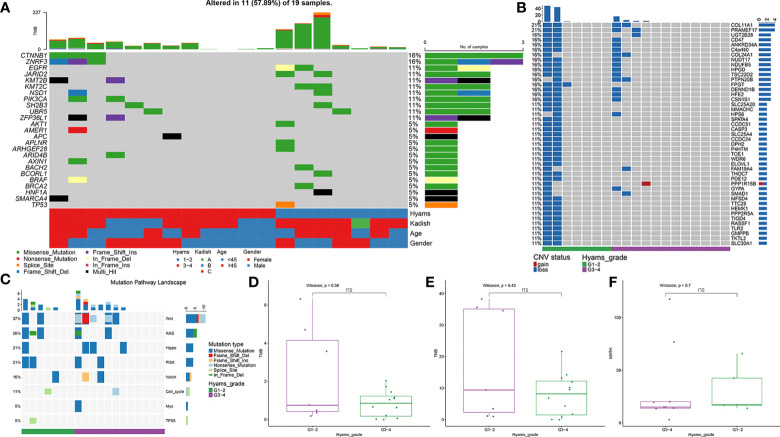
Mutational landscape of olfactory neuroblastoma (ONB). **(A)** Landscape of somatic mutations, including single nucleotide variants (SNVs) and small insertions and deletions. **(B)** Landscape of copy-number variations (CNVs). **(C)** Oncogenic pathway analysis of mutated genes. Comparisons of **(D)** tumor mutation burden (TMB), **(E)** tumor neoantigen burden (TNB), and **(F)** mutant-allele tumor heterogeneity (MATH) between the low- and high-grade ONB groups. ns, Not significant.

A total of 929 nonsynonymous alterations (mostly missense mutations) were identified in 18 (94.74%) ONB cases (**Table S2**). No somatic mutations were detected in one ONB patient. The top 30 mutant genes are shown in **Figure S1**. The most prevalent altered cancer-related genes were *CTNNB1* (16%) and *ZNRF3* (16%), followed by *EGFR* (11%), *JARID2* (11%), *KMT2B* (11%), *KMT2C* (11%), *NSD1* (11%), *PIK3CA* (11%), *SH2B3* (11%), and *UBR5* (11%) (**Figure 1A**). *TP53* mutation was detected in one ONB case (5%). CNVs, mainly copy-number loss, were observed in 52.63% (10/19) of ONB cases. Loss of *COL11A1* and *PRAMEF17* were observed in 21% (4/19) of cases (**Figure 1B**). The most mutated oncogenic pathways were the WNT and RAS pathways (**Figure 1C**). No cancer-related genes or pathways were found that had significantly different mutation frequencies between the low- and high-grade groups. The median TMB for all samples was 0.45 mutations/Mb, with a range of 0 to 3.25. No significant differences were observed between the low- and high-grade groups for TMB, TNB, or MATH (**Figures 1D–F**).

### The immune landscape of Chinese ONB patients

**Figure 2:**
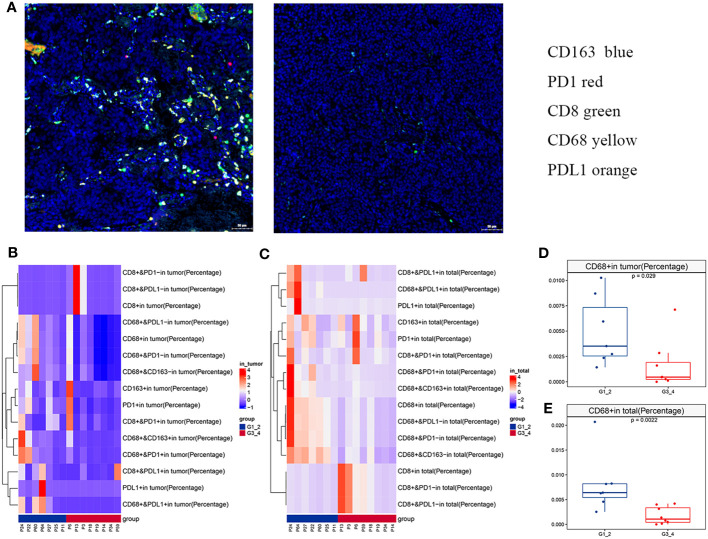
Immune landscape of olfactory neuroblastoma (ONB). **(A)** Representative multiplex immunofluorescence images of immunological markers, including CD8 (green), CD68 (yellow), CD163 (blue), PD-1 (orange), and PD-L1 (red). Heatmaps of immune cell infiltration in the **(B)** tumor region and **(C)** total region. The differences in CD68+ cells between the low- and high-grade groups in the **(D)** tumor region and **(E)** total region.

MIF assays were performed to examine immunological marker expression levels, including CD8, CD68, CD163, PD-L1, and PD-1, in the tumor, stroma, and total region (**Figure 2A**; **Table S3**). A total of 15 samples passed quality control, including seven low-grade and eight high-grade cases. Only one (1/15) case expressed PD-L1 (> 1%) in the tumor region. The percentage of CD8+ tumor-infiltrating lymphocytes (TILs) in the tumor region ranged from 0.03% to 84.9%, with a median of 1.08%. There was no difference in the percentage of CD8+ TILs between the low-grade and high-grade groups. However, the low-grade group showed significantly more CD68+ macrophages, including CD68+, CD68+CD163-, CD68+CD163+, CD68+PD-1-, CD68+PD-1+, CD68+PD-L1-, and CD68+PD-L1+ cells, in both the tumor and total region (**Figures 2B–E** and **Figure S2**) than the high-grade group. Notably, CD68+CD163- macrophages accounted for an average of 80.5% of the total CD68+ macrophages.

## Discussion

ONB is a rare sinonasal cancer with heterogeneous clinical behavior. It can range from an indolent form to rapidly growing disease that mainly metastasizes to the lymph nodes, brain, and lungs (28). The rarity of the disease has limited our understanding of its genomics and the development of individualized medicine. This is the first genomic study of ONB in China. We analyzed 19 ONB cases and found that 18 (94.74%) had a total of 929 nonsynonymous alterations (mostly missense mutations). The most prevalent altered cancer-related genes were *CTNNB1* (16%) and *ZNRF3* (16%). We also found that the low-grade group showed significantly more CD68+ macrophages than the high-grade group.

**Figure 3:**
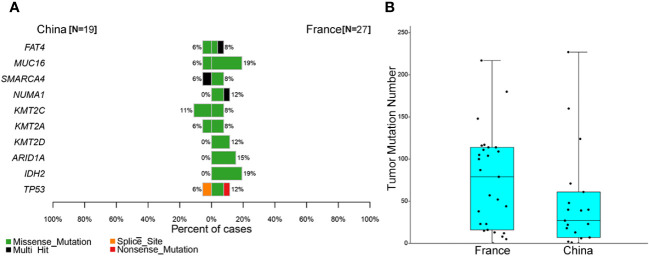
Comparison of somatic mutations between the Chinese and French olfactory neuroblastoma (ONB) cohorts. **(A)** Comparison of the top 10 cancer-associated genes in the French ONB cohort. **(B)** Comparison of the tumor mutation burden (TMB) between the ONB cohorts.

Few studies have explored the genomic landscape of ONB using different sequencing techniques. Cha et al. identified the TP53 missense mutation and the loss of CDKN2C in a metastatic ONB sample (9). Similarly, a recent comprehensive genomic study by Gay et al. revealed that the most commonly altered genes in ONB included TP53, PIK3CA, NF1, CDKN2A, and CDKN2C (23). In contrast to these studies, our data suggested that the most prevalent altered cancer-related genes were *CTNNB1* (16%) and *ZNRF3* (16%). *TP53* mutation was detected in one ONB case (5%) (7, 29, 30). Loss of *COL11A1* and *PRAMEF17* were observed in 21% (4/19) of cases. About 37% of ONB patients in this study had Wnt/β-catenin pathway abnormalities. Multiple genes within the Wnt/β-catenin pathway, including *CTNNB1* and *ZNRF3*, exhibited mutations in this cohort. Loss-of-function mutations in these genes lead to deregulated Wnt/β-catenin signaling and excessive stem cell renewal/proliferation, and are associated with metastatic disease (31, 32). A previous study in France showed that about 19% of ONBs harbor an *IDH2* R172 mutation (14). However, *IDH2* mutations were not detected in our cohort (**Figure 3**, 0 vs. 19%, P = 0.067). Moreover, there was no significant difference between our cohort and the French cohort for the frequency of mutations in all genes (**Figure 3A** and **Figure S3**). The median TMB value in our cohort was lower than that in French ONB patients, but not statistically significant (**Figure 3B**). These results imply that there may be differences in mutated genes in various ONB populations, demonstrating the need for further studies with more cases.

A previous study of 14 ONB cases showed that the TMB of the samples ranged from 1.3 to 9.6 mutations/Mb, with a mean of 3.8 mutations/Mb (33). Our study found the median TMB to be 0.45 mutations/Mb, with a range of 0 to 3.25 mutations/Mb. Consistent with the report by Friedman et al. (31), no association was observed between TMB and Hyams’ grade. As reported in few studies PD-L1 expression in ONB samples was found to be poor, which translates into a lower chance of response to anti-PD-1/PD-L1 drugs (12), consisted with previous studies, in our study only one (1/15) case expressed PD-L1 (combined positive score (CPS) ≥ 1) in the tumor region. When compared with non-small cell lung cancer and skin melanoma, cancer types in which immunotherapy has shown good response (34, 35), ONB samples had significantly less upregulation of PD-L1. The results of TMB, PD-L1, and CD8+ Tils suggest that ONB may be insensitive to immunotherapy. In this study, we found that the low-grade group showed significantly more CD68+ macrophages in both the tumor and total region compared with the high-grade group. This suggests that CD68-related macrophages may be associated with prognosis in low-grade ONB. Notably, CD68+CD163- macrophages accounted for an average of 80.5% of CD68+ macrophages, indicating that a low proportion of M1 macrophages in ONBs may be associated with poor prognosis. Tumor-associated macrophages (TAMs) are among the most prominent cellular components at the tumor microenvironment that have the potential to hinder the antitumor immune responses through the ignition of an immunosuppressive cytokine storm that facilitates tumor invasion and metastasis (36). An emerging therapeutic approach to overcome the TME barrier is to reprogram TME cells so that they assume an anti-tumor phenotype giving therapeutic agents a better fighting chance (37). The repolarization of TAMs from the pro-tumorigenic M2 into the anti-tumorigenic M1 phenotype has shown promising results in terms of tumor regression (38).

A 2019 study by Classe et al. evaluated the Ki-67 proliferation index as prognostic alternates to the current Hyams grading system. They demonstrated an association between a high Ki-67 proliferation index value and high-grade ONB (38). Our study also found significantly different Ki-67 expression levels between the low- and high-grade ONB groups, suggesting that high Ki-67 expression is associated with poor prognosis in ONB.

This study has some limitations. First, due to the short follow-up and the fact that most patients did not reach an event, we are not able to provide survival analyses for disease−free survival (DFS) and overall survival (OS). Second, due to the small number of samples in this study, the role of CD68 macrophages in ONB development still needs to be explored.

## Conclusion

Taken together, this study reports the genomic and immune landscape of Chinese ONB cases and compares the genomic features between low- and high-grade ONB. These data may be helpful for the individualized clinical management of ONB. *CTNNB1* and *ZNRF3* were the most prevalent altered cancer-related genes. The results of TMB, PD-L1, and CD8+ Tils suggest that ONB may be insensitive to immunotherapy. The low-grade group showed significantly more CD68+ macrophages in both the tumor and total region than the high-grade group. Notably, CD68+CD163- macrophages accounted for an average of 80.5% of CD68+ macrophages, indicating that M1 macrophages may play an important role in ONB development.

## Data availability statement

The authors acknowledge that the data presented in this study must be deposited and made publicly available in an acceptable repository, prior to publication. The datasets presented in this study can be found in online repositories. 

## Ethics statement

The studies involving humans were approved by Ethics Committee of Beijing Tongren Hospital (No. MR-11-23-000050). The studies were conducted in accordance with the local legislation and institutional requirements. The participants provided their written informed consent to participate in this study.

## Author contributions

YY: Conception and design, provision of study materials or patients, data collection, manuscript writing. ZW: Data analysis and interpretation, manuscript writing. EZ: Conception and design, data analysis and interpretation. YP: Manuscript writing. All authors contributed to the article and approved the submitted version.

## Supplementary material

Supplementary Figure 1 Mutational landscape of the top 30 genes in olfactory neuroblastoma (ONB).

Supplementary Figure 2 The differences in CD68+ cells between the low- and high-grade olfactory neuroblastoma (ONB) groups in the tumor region and total region.

Supplementary Figure 3 Comparison of the top 30 mutated genes between the Chinese and French olfactory neuroblastoma (ONB) cohorts.
