# Supplementary figures and images for: Genomic profiling and immune landscape of olfactory neuroblastoma in China
Source: Front Oncol. 2023 Nov 1;13:1226494. doi: 10.3389/fonc.2023.1226494 (PMC10646513; doi:10.3389/fonc.2023.1226494)

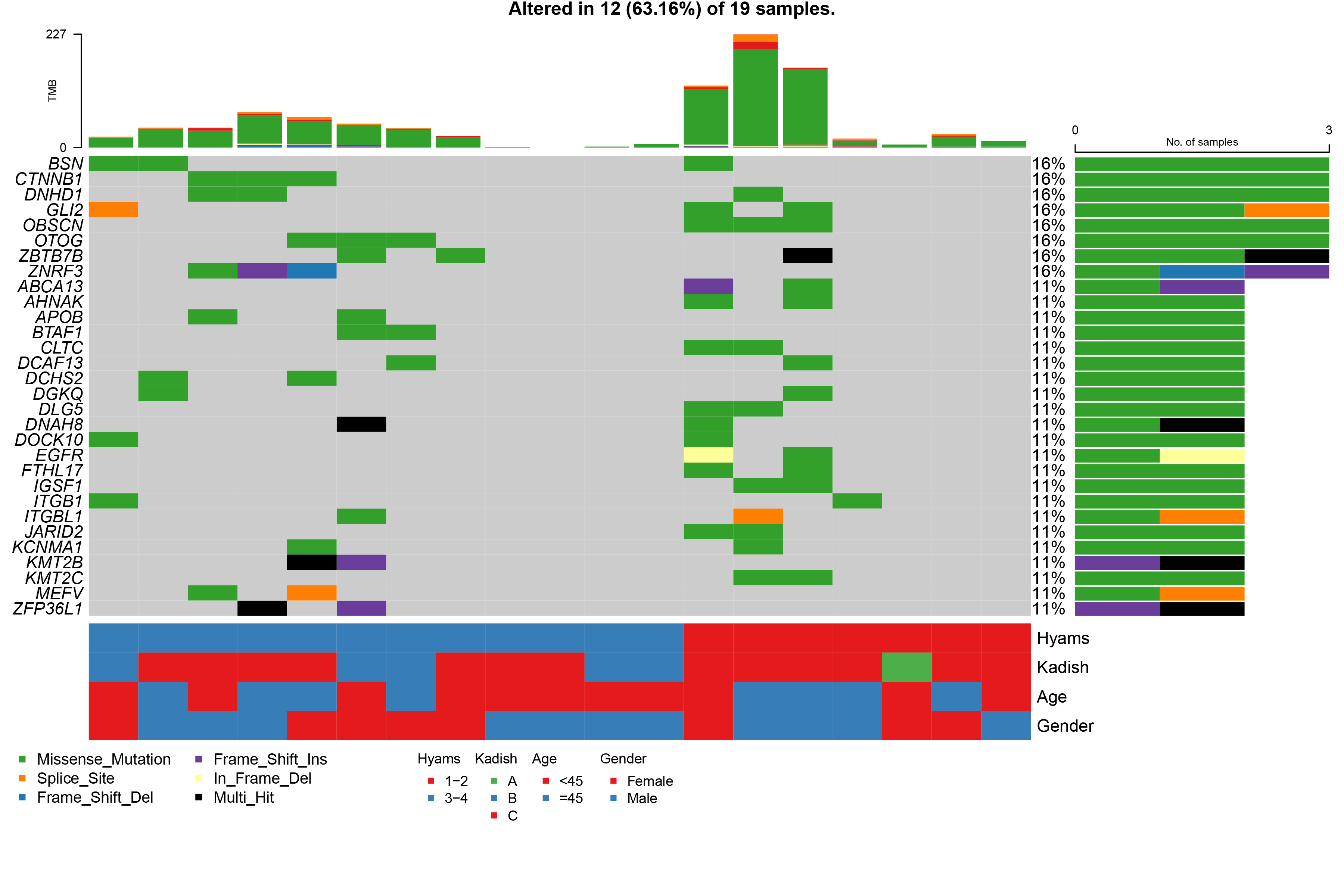

Supplement: Supplementary Figure 1 — Mutational landscape of the top 30 genes in olfactory neuroblastoma (ONB). [file Image_1.tif]

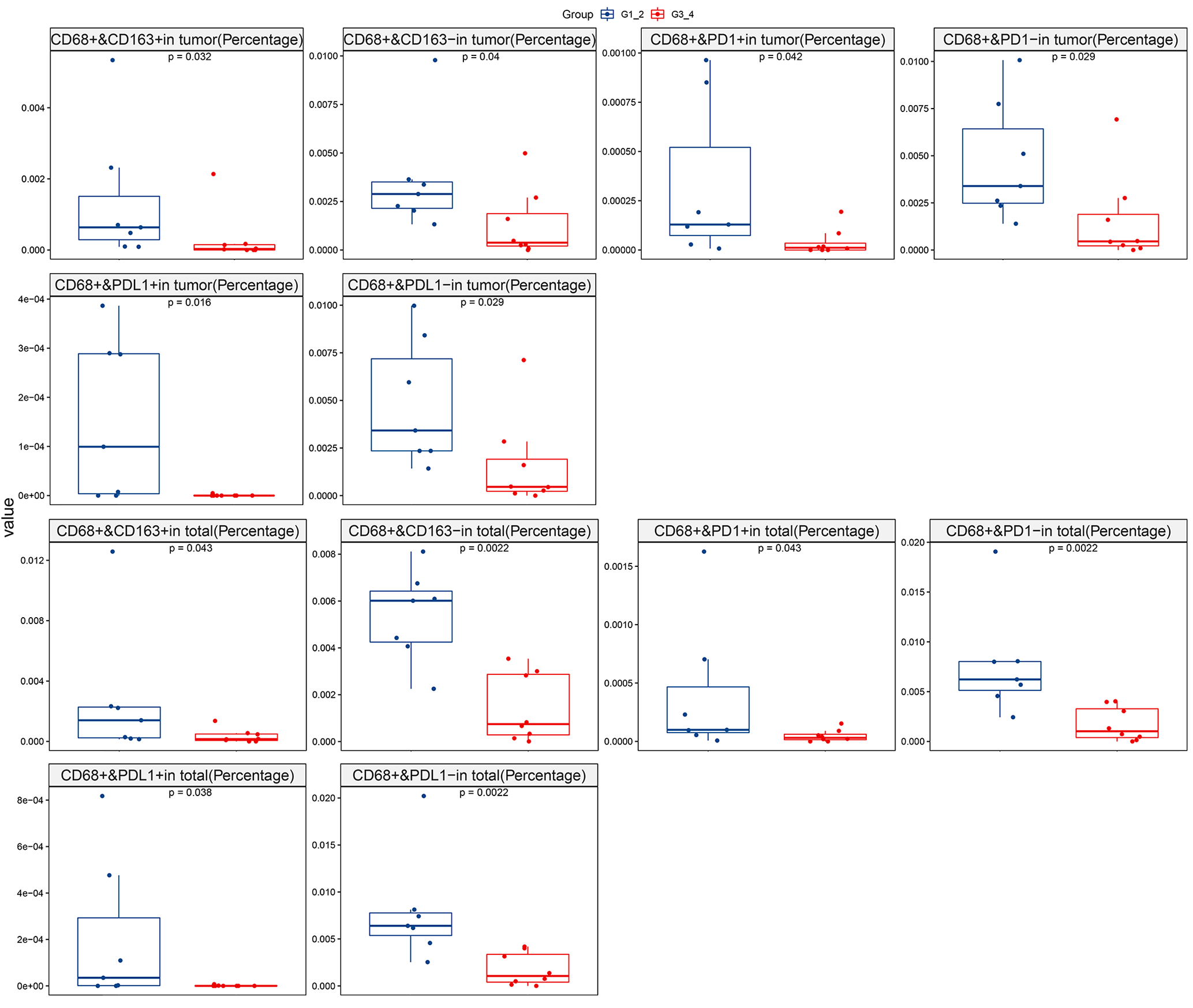

Supplement: Supplementary Figure 2 — The differences in CD68+ cells between the low- and high-grade olfactory neuroblastoma (ONB) groups in the tumor region and total region. [file Image_2.tif]

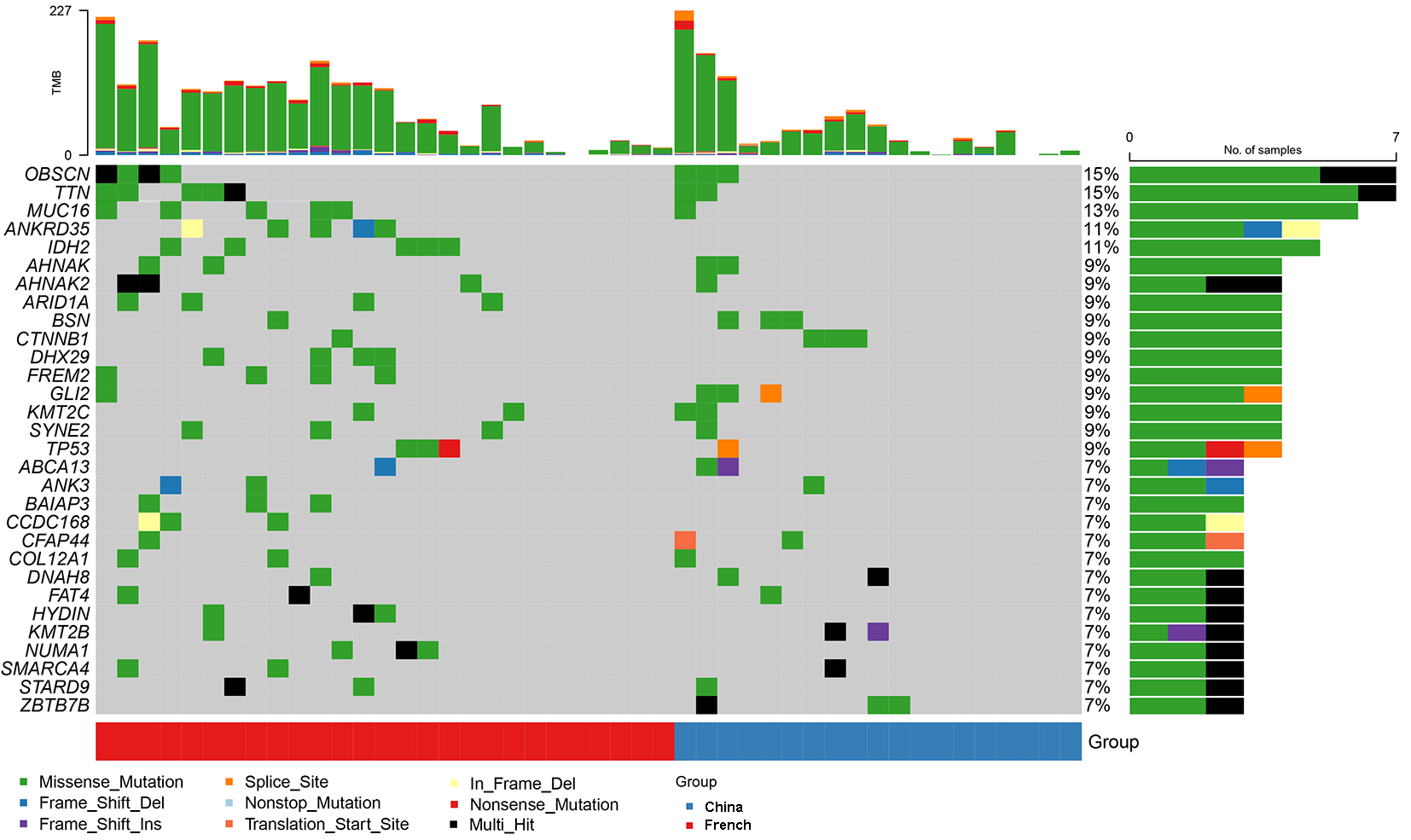

Supplement: Supplementary Figure 3 — Comparison of the top 30 mutated genes between the Chinese and French olfactory neuroblastoma (ONB) cohorts. [file Image_3.tif]
