# Supplementary material for: Genomic profiling and immune landscape of olfactory neuroblastoma in China
Source: Front Oncol. 2023 Nov 1;13:1226494. doi: 10.3389/fonc.2023.1226494 (PMC10646513; doi:10.3389/fonc.2023.1226494)
Supplement: Supplementary file 4 [file Table_1.docx]

**Supplemental table 1**

|  |  | Number  (percentage) |
| --- | --- | --- |
| **Gender** |  |  |
|  | Female | 8(42.11) |
|  | Male | 11(57.89) |
| **Kadish_stage** |  |  |
|  | A_B | 6(31.58) |
|  | C | 13(68.42) |
| **Hyams_grade** |  |  |
|  | G1_2 | 7(36.84) |
|  | G3_4 | 12(63.16) |
| **Age** |  |  |
|  | Mean(SD) | 42.3(13.3) |
|  | Median[Min,Max] | 42[22,72] |
| **First_treatment** |  |  |
|  | Surgery | 7(36.8) |
|  | Surgery+chemotherapy | 1(5.3) |
|  | Neoadjuvant_chemotherapy | 11(57.9) |
| **Chemotherapy** |  |  |
|  | Endostatin | 5(26.3) |
|  | Etoposide | 8(42.1) |
|  | Paclitaxel | 12(63.2) |
|  | Nedaplatin | 2(10.5) |
|  | Cisplatin | 10(52.6) |
| **Radiotherapy** |  |  |
|  | No | 17(89.5) |
|  | Yes | 2(10.5) |
| **Resection_margin** |  |  |
|  | R0 | 15(78.9) |
|  | R1 | 4(21.1) |
| **Recurrence** |  |  |
|  | No | 17(89.5) |
|  | Yes | 2(10.5) |
